# Congenital CMV infection in a Brazilian neonatal intensive care unit: high prevalence among twin newborns

**DOI:** 10.1186/s12985-024-02324-y

**Published:** 2024-03-08

**Authors:** Lauro Juliano Marin, Pérola Rodrigues dos Santos, Felipe Charu Ramos, Uener Ribeiro dos Santos, Marcílio Marques, Luciana Debortoli de Carvalho, Sandra Rocha Gadelha, Victor Hugo Aquino

**Affiliations:** 1 Laboratório de Farmacogenômica e Epidemiologia Molec ular, Departamento de Ciências da Saúde, Universidade Estadual de Santa Cruz, Ilhéus, Bahia Brasil; 2 Laboratório de Imunobiologia, Departamento de Ciências Biológicas, Universidade Estadual de Santa Cruz, Ilhéus, Bahia Brasil; 3 Laboratório de Microbiologia, Departamento de Ciências Biológicas, Universidade Estadual de Santa Cruz, Ilhéus, Bahia Brasil; 4 Faculdade de Medicina, Departamento de Ciências da Saúde, Universidade Estadual de Santa Cruz, Ilhéus, Bahia Brasil; 5 Departamento de Inmunología, Instituto de Investigaciones en Ciencias de la Salud, Universidad Nacional de Asunción, San Lorenzo, Central, Paraguay

**Keywords:** Cytomegalovirus, Congenital infection, Newborn, Neonatal intensive care unit

## Abstract

**Background:**

Cytomegalovirus (CMV) is one of the most important pathogens associated with congenital infection worldwide. Most congenital CMV-infected infants are asymptomatic at birth; however, some can develop delayed sequelae, especially hearing loss.

**Methods:**

This study aimed to investigate the prevalence of congenital CMV infection in a neonatal intensive care unit in a low-income region of Brazil. The objectives extended to identifying associated factors, assessing the clinical status of infected newborns, and undertaking a two-year follow-up to discern potential long-term consequences in the affected infants. This cross-sectional prospective study enrolled newborns up to three weeks of life requiring intensive medical care. We employed a convenience sampling method to include 498 newborns and 477 mothers in the study. Categorical variables underwent analysis employing Fisher’s exact test, whereas the examination of continuous variables involved the Mann‒Whitney test.

**Results:**

CMV DNA was detected in saliva/urine samples from 6 newborns (1.21%), confirming congenital infection. We noted a significantly greater incidence (OR: 11.48; 95% CI: 2.519–52.33; *p* = 0.0094) of congenital infection among twins (7.14%) than among nontwins (0.66%). The twin patients exhibited discordant infection statuses, suggesting that only one of the babies tested positive for CMV. Most of the infected children were born to mothers who initiated sexual activity at a younger age (*p* = 0.0269). Only three out of the six newborns diagnosed with CMV infection underwent comprehensive clinical assessments and received continuous follow-up until they reached two years of age. Only one of the children had weight and height measurements below the norm for their age, coupled with developmental delays.

**Conclusions:**

The prevalence of congenital CMV infection among newborns admitted to the NICU was low and similar to that in the general population. However, we found a significantly greater incidence of congenital CMV infection in twins than in singletons. Interestingly, the twin-infected patients exhibited discordant infection statuses, suggesting that CMV was present in only one of the babies. We also found that most of the infected children were born to mothers who initiated sexual activity at a younger age. Diagnostic accessibility and comprehensive surveillance programs are imperative for effectively managing and preventing congenital CMV infections.

**Supplementary Information:**

The online version contains supplementary material available at 10.1186/s12985-024-02324-y.

## Introduction

Cytomegalovirus (CMV), a member of the *Herpesviridae* family, is a widely distributed and prevalent agent of congenital infection, infection-related malformations, and neurological disabilities in developed and developing countries [1]. Mother-to-child transmission of congenital CMV infection can occur because of the mother’s primary infection, reinfection, or reactivation of the latent virus during pregnancy [2–6]. The transmission mechanism may contribute to the complex dynamics of congenital infection and negatively impact the outcomes of affected infants. Maternal primary infection is associated with a higher risk of transmission (approximately 40% of fetuses with symptomatic presentation), while viral reactivation or reinfection by another strain has an infection rate of only 2% for fetuses [7].

Congenital CMV infection can manifest at any stage during gestation, with a greater risk of infection occurring in the first trimester, the most critical period of embryonic development [4, 8, 9]. While most newborns infected with congenital CMV may not exhibit any visible symptoms, approximately 10% of cases are symptomatic and present a range of signs and symptoms. These symptomatic patients can present with a range of clinical manifestations, including petechiae, hepatosplenomegaly, jaundice, and neuromotor and sensory impairments [10–13]. Neurological sequelae are particularly significant in symptomatic patients, with infants often experiencing microcephaly and hearing deficits. These impairments can extend beyond infancy, affecting the child’s cognitive development, language acquisition, and overall quality of life [1].

In addition to the potential long-term consequences in affected infants, congenital CMV infection may lead to complications during the gestational period, including intrauterine growth retardation and low birth weight. Additionally, infants born prematurely with congenital CMV infection may exhibit symptoms at birth, further highlighting the impact of this viral infection on both fetal and neonatal health [14, 15]. Furthermore, even newborns with congenital CMV infection who are asymptomatic are at risk of developing sequelae that may manifest later in life. Congenital CMV infection is a significant non-genetic cause of hearing loss and delayed neurocognitive development in children [16–18]. Indeed, the differential and early diagnosis of congenital CMV infection are crucial for effective disease management and prevention of long-term sequelae [19, 20].

Congenital CMV infection exhibits a higher prevalence in developing nations compared to developed ones, impacting an estimated 1 to 5% of births [21]. In the United States, an estimated 20,000 to 30,000 cases of congenital CMV infection in newborns are reported annually [1]. In Brazil, the prevalence of congenital CMV ranges from 1.08 to 1.19% [22, 23]. Intriguingly, some investigations have shown an increase in the occurrence of congenital CMV infection among newborns requiring medical intensive care, with rates of 6.8% in Brazil and 10% in Japan [24, 25]. These findings suggest that congenital cytomegalovirus (CMV) infection may significantly contribute to neonatal health complications. This fact could explain the observed higher incidence of CMV infection among newborns in NICUs, highlighting the importance of understanding and addressing the impact of CMV in this vulnerable population [26].

This study aimed to investigate the prevalence of congenital cytomegalovirus (CMV) infection in the neonatal intensive care unit (NICU) of a low-income region of Brazil. The objectives extended to identifying associated factors, assessing the clinical status of infected newborns, and undertaking a two-year follow-up to discern potential long-term consequences in the affected infants.

## Materials and methods

### Study design

This cross-sectional prospective study was conducted at Manoel Novaes Hospital (MNH) in Itabuna, Bahia State, Brazil, from May 2014 to December 2019. This hospital specializes in emergency and elective outpatient care for pediatric, obstetric, and oncological patients at a secondary level. The hospital also serves as a high-risk neonatology reference center, attending 120 municipalities in southern Bahia State. The hospital manages around 350 pregnant mothers monthly, translating to approximately 4,200 patient-years of attention. The NICU of this hospital manages an average of 40 to 50 infants every month, resulting in a yearly range of 480 to 600 newborns receiving specialized care.

We determined the sample size for this study based on the reported prevalence of 6.8% in NICUs of Minas Gerais state [24], which shares proximity with Bahia state. Using the GRANMO software (Institut Municipal d’Investigació Mèdica, Barcelona, Spain) and considering an alpha risk of 0.05 and a beta risk of 0.2 in a two-sided test, we calculated that 247 subjects were required for the observed group to detect a difference equal to or greater than 0.05 units. The proportion in the reference group was estimated to be 0.068, and a dropout rate of 5% was anticipated.

We used a convenience sampling approach to select our study participants, primarily due to the considerable geographical distance separating the hospital and the university where the research team is based.

### Study population

The eligible participants were newborns admitted to the NICU for any reason and their mothers. Mothers who willingly participated in the study received a comprehensive informed consent form. They were encouraged to review the document carefully and ensure a clear understanding of the study before collecting any biological samples or medical records. The participant’s data were thoroughly de-identified for complete anonymity to protect privacy and confidentiality.

The inclusion criterion encompassed newborns up to three weeks of age receiving care in the NICU and their mothers. The exclusion criteria involved children for whom collecting a clinical sample was not feasible or whose clinical and/or epidemiological data were incomplete.

We collected maternal sociodemographic data and clinical information concerning the newborns at birth using a semistructured questionnaire administered to the mothers and data from their medical records.

### Sample collection

Upon recruitment and when the children return for clinical evaluation, they have saliva and/or urine samples collected. We collected saliva samples using a sterile swab (Labor Import, Brazil) inserted into the newborn’s mouth, which was gently and circularly rotated for approximately one minute, after which the swab was transferred to a sterile plastic microtube (Eppendorf, 2 mL) containing 600 µL of Earle MEM transport medium (Cultilab, Brazil). Urine samples were collected aseptically using a hypoallergenic universal collection bag (Cral Plast, 100 mL), taking care to avoid contamination with meconium. The biological samples were transported under refrigeration to the Laboratório de Farmacogenômica e Epidemiologia Molecular (LAFEM) at the Universidade Estadual de Santa Cruz and stored in a freezer at -20 °C until further processing.

### Laboratory diagnosis of congenital CMV infection

The viral genetic material was identified using a two-step polymerase chain reaction (nested PCR), as previously described [23]. The PCR products were subjected to 2% agarose gel electrophoresis using 10 µL of each amplified product mixed with GelRed nucleic acid dye (Biotium, USA). Electrophoresis was performed using Tris/Borate/EDTA buffer solution (0.089 M Tris, 0.089 M borate, 0.01 M EDTA, pH 7.5) and run at 150 V for 30 min. The agarose gel was photographed using a digital camera attached to a UV transilluminator (Loccus, Brazil). Every essential precaution was meticulously taken to eliminate the possibility of cross-contamination during the nested PCR test. Participants who tested positive on PCR provided an additional sample. Confirmation of CMV infection in newborns relied on the positive PCR results obtained from two consecutive samples (saliva or urine) collected within three weeks of birth.

### Clinical evaluation of children with congenital CMV infection

Newborns with confirmed congenital CMV infections underwent posthospitalization medical assessments led by MNH pediatricians, typically scheduled until the child completed two years of age. These evaluations encompassed various aspects, such as gathering anthropometric data (weight, height, head circumference) and conducting thorough clinical examinations to identify potential sequelae resulting from the infection. Moreover, otorhinolaryngologists assessed the children’s hearing abilities and determined the degree of auditory acuity through brainstem auditory evoked potential (BAEP) testing to evaluate neurological deafness.

### Statistical analysis

This study investigated the correlation between each reported epidemiological and clinical variable and the detection of CMV in newborns. Categorical variables were analyzed using Fisher’s exact test, while normality testing with the Kolmogorov‒Smirnov test preceded the statistical analysis of continuous data. Continuous variables were analyzed using the Mann‒Whitney test. The data is presented as absolute numbers (n), percentages (%), medians, or interquartile ranges (IQRs) where applicable. We determined the statistical significance among the groups with a significance level (alpha) set at 0.05.

## Results

**Fig. 1:**
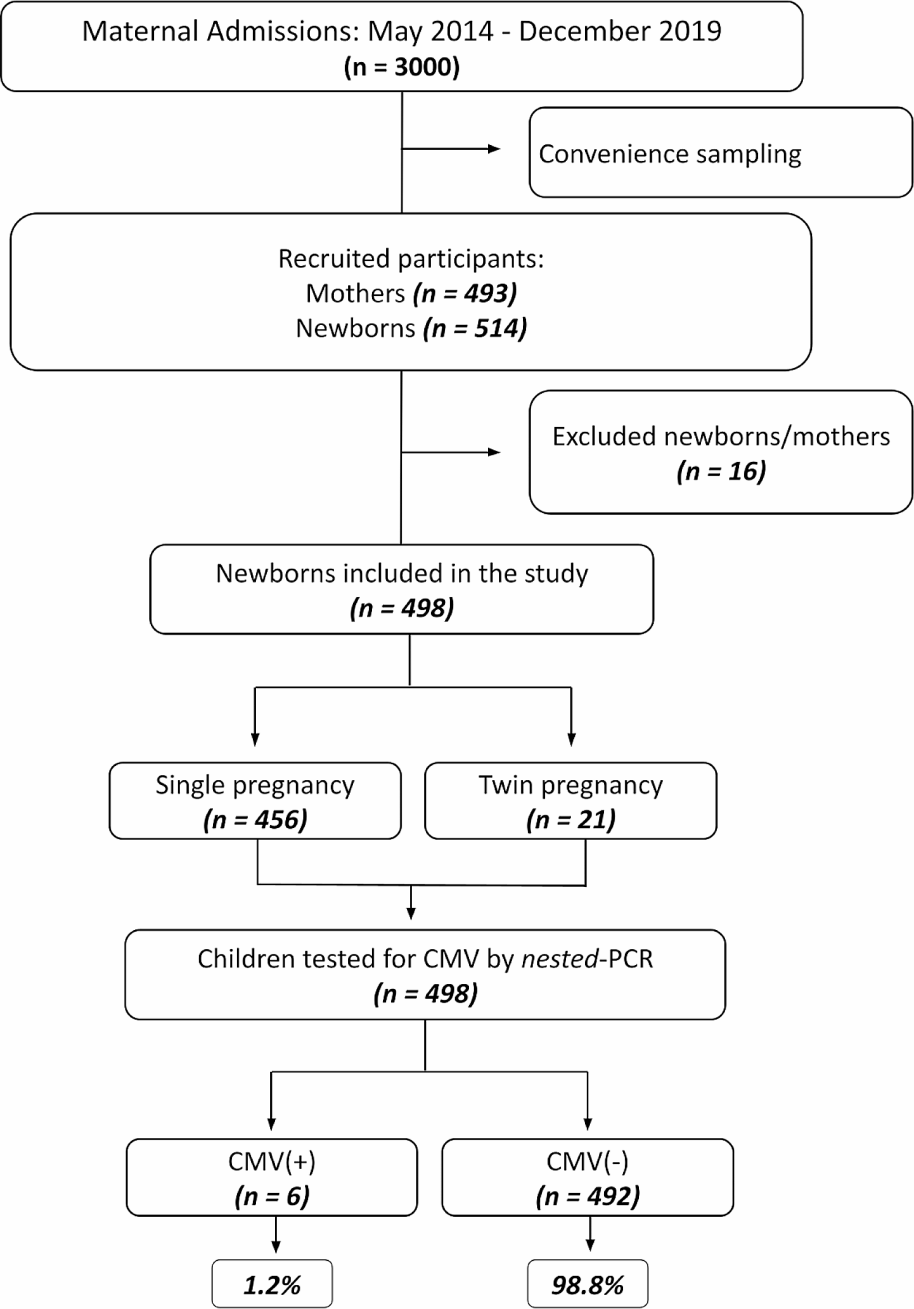
Diagram showing the flow of participants through the study

From May 2014 to December 2019, the NICU provided medical care to approximately 3,000 newborns. We selectively enrolled 514 newborns, representing 493 mothers, through a convenience sampling methodology. However, we excluded 16 newborns with their respective mothers from the analysis because of the lack of biological sample collection or incomplete clinical or epidemiological data. The study included 498 newborns from 477 mothers (21 of whom had twin births) (Fig. 1, Supplementary Table S1).

The mothers in this study had a median age of 26 years (Q1 20 - Q3 32), and the newborns had a median age of 6 days (Q1 2 - Q3 14). Table 1 shows a comprehensive overview of the study population characteristics.

**Table 1:** Characteristics of the study population

Mother	Median	Q1 - Q3
**Age, years**	26	20–32
**Age at the first intercourse, years**	16	15–18
**Sexual partners**	1	1–3
**Number of children**	1	1–2
**Number of consultations**	7	5–8
	**n**	**%**
**Type of delivery**		
Cesarian section	267	53.61
Normal delivery	227	45.59
Not related	4	0.80
**Condom use**		
Yes	132	26.51
No	331	66.46
Not related	35	7.03
**Contraceptive use**		
Yes	229	45.98
No	252	50.60
Not related	17	3.42
**Medicine use**		
Yes	111	22.29
No	366	73.49
Not related	21	4.22
**CMV knowledge**		
Yes	93	18.67
No	398	79.92
Not related	7	1.41
**Maternal occupation**		
Yes	408	81.83
No	7	1.41
Not related	83	16.66
**Twins**		
Yes	42	8.43
No	456	91.57
**Newborn**		
	**n**	**%**
**Sex**		
Female	243	48.80
Male	255	51.20
	**Median**	**Q1 -Q3**
**Weight, kg**	2.349	1.633–3.030

**Fig. 2:**
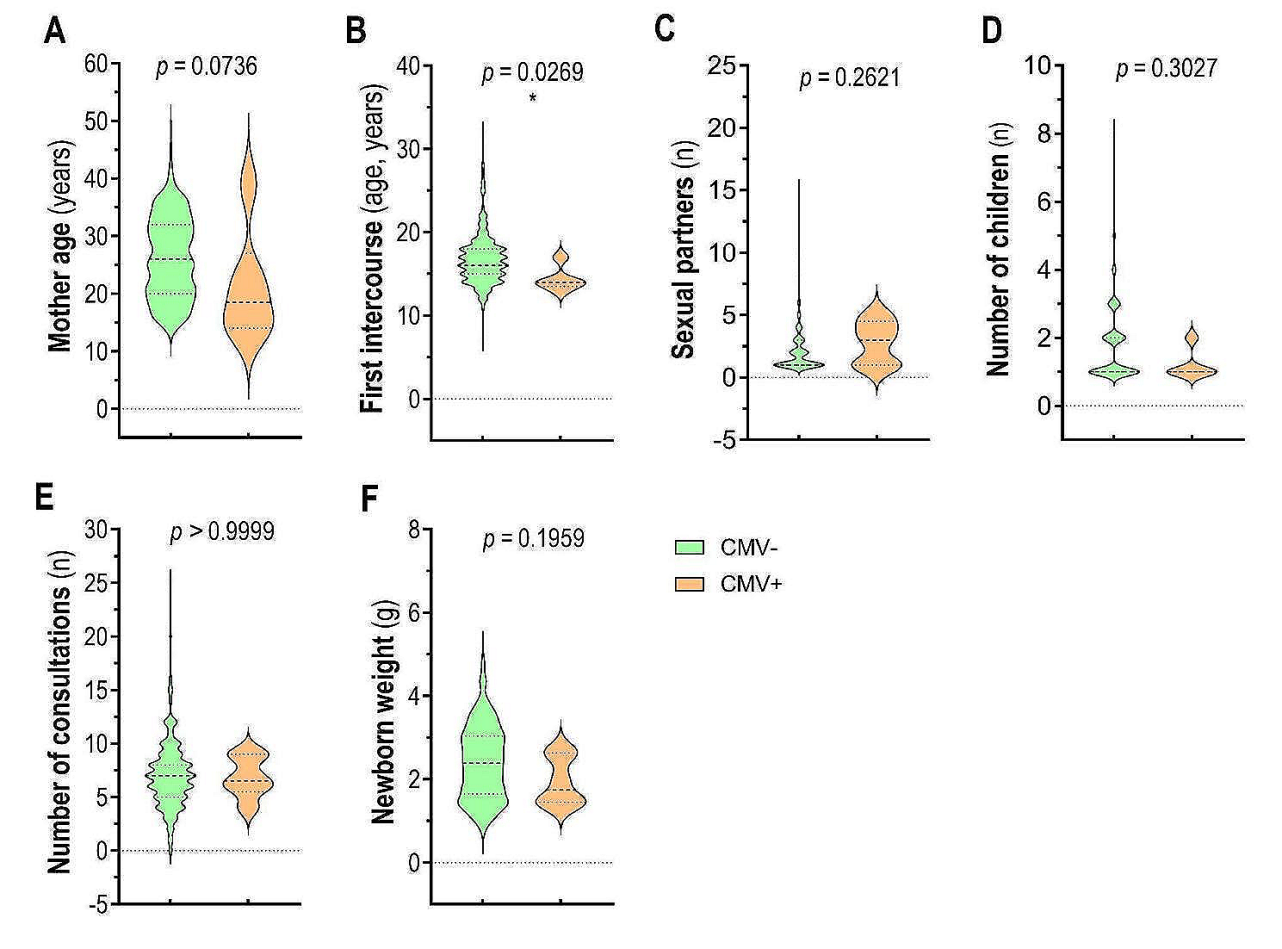
Comparison of maternal and newborn characteristics based on CMV infection status. Maternal characteristics, encompassing (**A**) age, (**B**) age at first intercourse, (**C**) number of sexual partners, (**D**) number of children, and (**E**) the number of consultations before delivery, as well as child characteristics, and specifically (**F**) newborn weight, are depicted through violin plots. The thick-dotted line indicates the median values, while the thin-dotted lines indicate the interquartile range (IQR). The statistical significance of the differences in patient characteristics was determined using the Mann‒Whitney test, with p-values less than 0.05 considered to indicate statistical significance

PCR confirmed congenital CMV infection in 6 (1.2%) newborns. Most of the infected children (OR: 0.1669; 95% CI: 0.008–3.384 *p* = 0.0094) were born to mothers who initiated sexual activity at a younger age (Fig. 2B). Newborns from twin pregnancies were more likely to be positive for congenital CMV infection (OR: 11.48; 95% CI: 2.519–52.33; *p* = 0.0094) than newborns from nontwin pregnancies were (Table 2).

**Table 2:** Clinic and epidemiological characteristics of mothers and newborns according to CMV infection status

Variables	Total child (*n* = 498)	Mother		OR	95% IC	p values
		CMV (+) child (*n* = 6)	CMV (-) child (*n* = 492)			
**Type of delivery**	**n (%)**	**n (%)**	**n (%)**			
Cesarian section	267 (53.62)	3 (0.60)	264 (53.02)	0.8523	0.197–3.680	*> 0.9999*
Normal	228 (45.78)	3 (0.60)	225 (45.18)			
Unknown*	3 (0.60)	0 (0.00)	3 (0.60)			
**Condom use**						
Yes	132 (26.50)	1 (0.20)	131 (26.30)	0.8302	0.129–5.327	*> 0.9999*
No	331 (66.47)	4 (0.80)	327 (65.66)			
Unknown*	35 (7.03)	1 (0.20)	34 (6.83)			
**Contraceptive use**						
Yes	229 (45.98)	1 (0.20)	228 (45.78)	0.2954	0.048–1.813	*0.2190*
No	252 (50.60)	5 (1.00)	247 (49.60)			
Unknown*	17 (3.42)	0 (0.00)	17 (3.42)			
**Medicine use**						
Yes	111 (22.29)	0 (0.00)	111 (22.29)	0.000	0.000-2.327	*0.3439*
No	366 (73.49)	6 (1.20)	360 (72.29)			
Unknown*	21 (4.22)	0 (0.00)	21 (4.22)			
**CMV knowledge**						
Yes	93 (18.67)	1 (0.20)	92 (18.47)	1.160	0.188–7.157	*> 0.9999*
No	398 (19.92)	5 (1.00)	393 (78.92)			
Unknown*	7 (1.41)	0 (0.00)	7 (1.41)			
**Maternal Occupation**						
Yes	408 (81.93)	4 (0.80)	404 (81.13)	0.1669	0.008–3.384	*> 0.9999*
No	7 (1.40)	0 (0.00)	7 (1.40)			
Unknown*	83 (16.67)	2 (0.40)	81 (16.67)			
**Newborn sex**						
Female	243 (48.80)	2 (0.40)	241 (48.39)	0.5786	0.122–2.743	*0.6862*
Male	255 (51.20)	4 (0.80)	251 (50.40)			
**Twins**						
Yes	42 (8.43)	3 (0.60)	39 (7.83)	11.48	2.519–52.33	***0.0094***
No	456 (91.57)	3 (0.60)	453 (90.96)			

OR, odds ratio. 95% CI, confidence interval.^a^ Fisher’s exact test. Values of *p* < 0.05 were considered statistically significant. Bold values indicate statistical significance.*Not included in statistical analysis

**Fig. 3:**
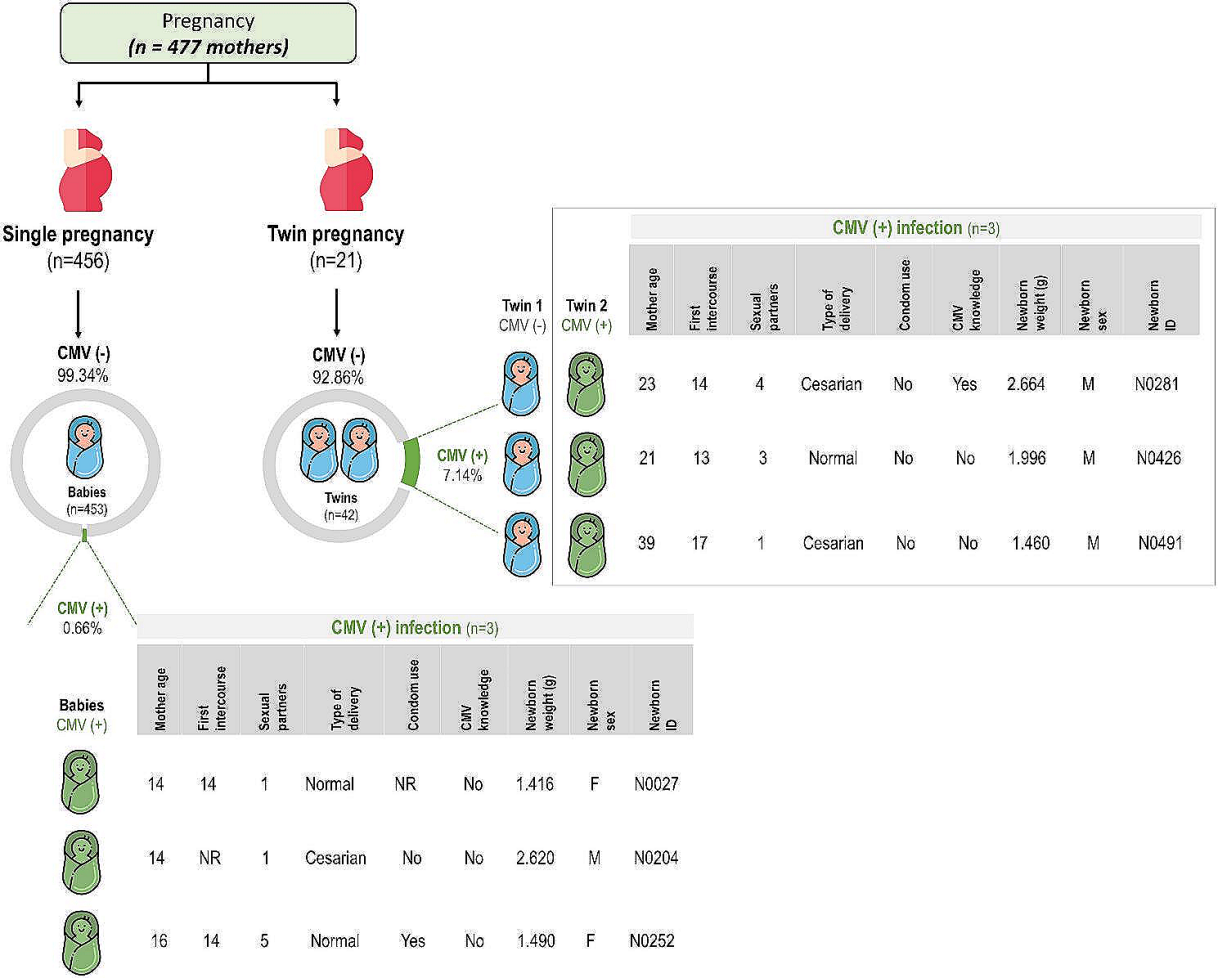
Characteristics of the twins and their mothers. The figure was created with freely available images (ww.flaticon.com)

Among the newborns infected with CMV, half (3 out of 6) were twins. Interestingly, only one of the twins in each pair was infected. Figure 3 illustrates the characteristics of the twins and their mothers.

Only three out of the six newborns diagnosed with CMV infection underwent comprehensive clinical assessments and received continuous follow-up until they reached two years of age. The remaining patients did not receive clinical evaluation because they moved to another region or did not comply with scheduled medical follow-ups.

Among the three infants clinically evaluated, 2 had growth and developmental parameters within the appropriate standards for their age, exhibiting no discernible physical indications of hepatic, visual, or auditory impairment. The audiometry tests yielded results within the normal range, and the BAEP test indicated no evidence of hearing loss. Conversely, one of the assessed infants had weight and height measurements below the norm for their age, coupled with developmental delays. During the auditory assessment, this child presented excessive earwax in the ear canal, necessitating wax removal before the test. Regrettably, the infant did not return for the procedure, rendering the evaluation of auditory sequelae impossible. Furthermore, two of the three infants who underwent clinical evaluation continued to excrete the virus until they reached two years of age, and one presented symptomatic manifestations.

## Discussion

In our study, we observed a prevalence of congenital CMV infection of 1.2% among newborns receiving NICU care at HMN. This result aligns closely with findings from neonatal screening studies conducted in low-income populations from public hospitals in Brazil outside of NICU settings [22, 23], suggesting that congenital CMV infection may not be a primary factor necessitating intensive care for newborns. Our identified prevalence is also consistent with studies in developing countries, such as China (1.32%) and Mexico (1.48%) [27, 28]. Furthermore, a systematic review exploring the prevalence of congenital CMV infection in developing countries reported rates of 1.8% in Chile, 0.9% in Mexico, and 1.8% in Taiwan [18]. However, the incidence of congenital CMV infection is lower in developed countries, for example, 0.3% in Japan and 0.2% in Finland. This discrepancy suggests an inverse relationship between the incidence of congenital CMV infection and socioeconomic status [29, 30].

While the general prevalence of congenital cytomegalovirus (CMV) infection typically remains below 2%, previous studies conducted in Brazil and Japan have reported higher rates ranging from 6.8 to 11.6% among newborns under NICU management [24, 25]. The discrepancies observed between the results found in our study and these earlier investigations in Brazil and Japan could be attributed to biases stemming from limited sample sizes and unclear sampling methodologies in the referenced studies. The Japanese population relies on a modest sample size of only 60 urine samples without comprehensive details about the sampling method. Likewise, the preceding Brazilian study, which included 292 newborns purportedly selected randomly, lacked details regarding the methodology employed for the randomization process. These methodological gaps highlight the importance of transparent reporting and robust study designs for ensuring the credibility and comparability of research outcomes.

A limitation of our study stems from the convenience sampling method, which potentially introduces bias into the obtained results. A random selection approach of participants would have been more appropriate. Nevertheless, we maintain that our findings offer a close approximation of the actual scenario, as we recruited, at regular intervals throughout the study period, a significant number of participants (*n* = 514), double the calculated sample size, *n* = 247, representing almost 17% of the newborns in NICU care.

Another limitation in our study pertained to the lack of information regarding the CMV IgG status of the mothers. The relatively low rate of congenital infection identified could potentially stem from the overall low prevalence of CMV infection among the pregnant women included in our study cohort. However, existing literature has consistently demonstrated a high prevalence of CMV IgG among pregnant women in developing nations, with rates surpassing 95% [31]. In a study conducted in 2018 within the same geographical region, we reported a CMV IgG prevalence of 95.2% among pregnant women [32]. These data underscore the reliability of our findings regarding the incidence of congenital CMV infection.

Our investigation revealed that mothers of CMV-infected newborns were younger at sexual debut than mothers of noninfected newborns. This observation is consistent with the results of a prior study by Fowler and Pass (2006) [33], which suggested that the initiation of sexual activity within two years before delivery represents a risk factor for congenital CMV infection. This elevated risk may be linked to early exposure to CMV, potentially resulting in viral infection and an augmented likelihood of CMV transmission, as discussed in the study by Raynor et al. (2022) [34].

We observed a greater incidence of congenital CMV infection in newborns from twin pregnancies (7.14%, *n* = 3/42) than in those from nontwin pregnancies (0.66%, *n* = 3/456). However, a further study with a higher number of twins is required to confirm this finding. Anyway, this result is consistent with findings from prior studies. For instance, a study conducted in Turkey reported an elevated incidence of congenital CMV infection in twin pregnancies (16.7%, *n* = 3/18) compared to nontwin pregnancies (1.32%, *n* = 12/908) [35]. Furthermore, research by Hutton and Rowan (2021) [36] based on published data investigated congenital CMV infection in newborns from mothers with primary infections during pregnancy, revealing a greater rate of vertical CMV transmission in multiple pregnancies (58.7%, *n* = 27/46) than in singleton pregnancies (31.4%, *n* = 429/1365).

Our findings revealed CMV infection in only one twin in each pair. Despite sharing the same maternal environment and possessing a similar genetic background, twins demonstrated varying responses to maternal infection, displaying a phenomenon consistent with our study and corroborated by earlier research [36]. The potential mechanisms contributing to this discordant infection may be associated with various factors, such as the type of chorion and placenta, along with disparities in immune status [37]. Regrettably, our analysis lacked specific data about these factors.

We performed clinical assessments on half of the children diagnosed with congenital CMV, specifically evaluating three out of six (50%). One of these patients displayed distinct clinical features, such as low weight and short stature appropriate for his age. In contrast, the other two children exhibited development within the normal range until they reached the age of two. A further limitation of our study was the relatively small number of children included in the follow-up analysis. These findings align with the literature, which reports that approximately 90% of infants with congenital CMV are asymptomatic at birth but that 10 to 15% of these infants may develop late-onset hearing loss [38]. The extended viral shedding observed in two children, while not linked to neurological developmental delay or growth disorders, underscores the elevated transmissibility of CMV and its persistence within the general population [39]; emphasizing the significance of assessing newborns until they reach the age of 2 by a pediatrician and an otolaryngologist is crucial, given the potential risk of late sequelae. The absence of symptoms in infants and the lack of awareness among mothers about the infection may have contributed to the suboptimal attendance at follow-up appointments. Our earlier investigation underscored a comparable trend, revealing a noteworthy lack of mothers’ adherence to scheduled medical follow-ups for their children [40]. In addition, the COVID-19 pandemic restricted access to the NICU and impeded proactive outreach to mothers residing in different municipalities, complicating clinical follow-up efforts.

The findings from this study underscore the critical role of health education as a pivotal tool for infection prevention and control, especially concerning congenital infections. Alarmingly, only 19.05% of mothers reported having any knowledge about CMV. Consequently, there is a pressing need to allocate increased resources to health education programs.

The absence of routine laboratory tests for diagnosing congenital CMV infection in numerous countries, including Brazil, has contributed to undetected cases, hampering timely intervention. The need for robust epidemiological serosurveillance programs further hinders prevention efforts and data collection. Enhancing diagnostic accessibility and instituting comprehensive surveillance programs are imperative for effectively managing and preventing congenital CMV infections.

## Conclusion

The prevalence of congenital CMV infection among newborns admitted to the NICU of a public hospital in southern Bahia, Brazil, was low (1.21%) and similar to that in the general population. However, we found a greater incidence of congenital CMV infection in twins than in singletons. Interestingly, the twin-infected patients exhibited discordant infection statuses, suggesting that CMV was present in only one of the babies. We also found that most of the infected children were born to mothers who initiated sexual activity at a younger age. Diagnostic accessibility and comprehensive surveillance programs are imperative for effectively managing and preventing congenital CMV infections.

### Electronic supplementary material

Below is the link to the electronic supplementary material.

Supplementary Material 1

## Data Availability

All relevant data are within the manuscript and its Supporting Information files.

## Abbreviations

CMV: Cytomegalovirus
NICU: neonatal intensive care unit
PCR: polymerase chain reaction
BAEP: brainstem auditory evoked potential
IQRs: interquartile ranges
OR: odds ratio
